# Chitosan Sponges with Instantaneous Shape Recovery and Multistrain Antibacterial Activity for Controlled Release of Plant-Derived Polyphenols

**DOI:** 10.3390/ijms24054452

**Published:** 2023-02-23

**Authors:** Ioana-Victoria Platon, Claudiu-Augustin Ghiorghita, Maria Marinela Lazar, Irina Elena Raschip, Maria Valentina Dinu

**Affiliations:** Department of Functional Polymers, “Petru Poni” Institute of Macromolecular Chemistry, Grigore Ghica Voda Alley 41A, 700487 Iasi, Romania

**Keywords:** chitosan, sponges, curcumin, shape recovery, antibacterial activity, controlled drug release

## Abstract

Biomass-derived materials with multiple features are seldom reported so far. Herein, new chitosan (CS) sponges with complementary functions for point-of-use healthcare applications were prepared by glutaraldehyde (GA) cross-linking and tested for antibacterial activity, antioxidant properties, and controlled delivery of plant-derived polyphenols. Their structural, morphological, and mechanical properties were thoroughly assessed by Fourier-transform infrared (FTIR) spectroscopy, scanning electron microscopy (SEM), and uniaxial compression measurements, respectively. The main features of sponges were modulated by varying the CS concentration, cross-linking ratio, and gelation conditions (either cryogelation or room-temperature gelation). They exhibited complete water-triggered shape recovery after compression, remarkable antibacterial properties against Gram-positive (*Staphylococcus aureus* (*S. aureus*), *Listeria monocytogenes (L. monocytogenes*)) and Gram-negative (*Escherichia coli* (*E. coli*)*, Salmonella typhimurium* (*S. typhimurium*)) strains, as well as good radical scavenging activity. The release profile of a plant-derived polyphenol, namely curcumin (CCM), was investigated at 37 °C in simulated gastrointestinal media. It was found that CCM release was dependent on the composition and the preparation strategy of sponges. By linearly fitting the CCM kinetic release data from the CS sponges with the Korsmeyer–Peppas kinetic models, a pseudo-Fickian diffusion release mechanism was predicted.

## 1. Introduction

Numerous pharmaceuticals, synthesized nowadays at the industrial level, were first extracted from plants, the most representative being salicin and quinine, isolated from the bark of *Salix alba* and *Cinchona succirubra*, respectively. Along with the two industrial revolutions, emphasis was placed on the use of compounds at a large scale obtained via synthetic methods due to increased efficiency, and less on the traditional use of plants, which have higher costs. However, the current trend is to return to the study of plant products, using the technological experience gained in the last two centuries to obtain formulations that correspond to the requirements imposed by the XXI century.

Phenolic compounds are a major class of metabolites, widely present in all vascular plants, containing at least one phenolic group in their structure. Plant-derived polyphenols are well known as strong antioxidants, which protect the human body against oxidative stress, premature tissue aging, and various chronic diseases (cardiovascular, cancer, inflammation, etc.) [1,2]. Due to their antioxidant and antimicrobial properties, they are also used as preservatives and bacteriostatics in food storage, for the control and preservation of food freshness, plus evaluation of the quality and safety of various agricultural products [1,2]. In particular, CCM, which is a yellow-orange polyphenol and the active ingredient of turmeric (*Curcuma longa*), is intensively investigated due to its pharmacological properties, such as the antioxidant, anti-inflammatory, anticholesterolemic, antihepatotoxic, anticarcinogenic, and neuroprotective features [1,2,3]. Despite the health benefits of CCM, this bioactive compound is poorly soluble in water (0.011 μg/mL at pH 5 and 0.4 μg/mL at pH 7.4) and unstable under heat, light, enzymes, and oxygen [3]. Furthermore, the clinical application of CCM is greatly limited by low absorption, rapid metabolism, and fast systemic elimination [1,2,3]. Consequently, developing delivery platforms with a high therapeutic efficacy for controlled release of CCM is still prevailing. Nevertheless, encapsulation can be an effective approach to preserve CCM characteristics and to safely deliver CCM to the targeted sites [4,5,6,7,8,9,10,11,12,13].

Polysaccharides, due to their biocompatibility, biodegradability, abundance, and adequate functional groups, are ideal matrices for encapsulation of different bioactive compounds, including CCM [13,14,15,16,17]. Among polysaccharides, CS, a cationic polymer comprising of N-acetyl-D-glucosamine and D-glucosamine units, produced by the partial deacetylation process of chitin, has drawn the attention of many scientists due to its intrinsic peculiarities, such as biodegradability, hemocompatibility, mucoadhesivity, anti-inflammatory, antibacterial, antioxidant, and hemostatic activities [18,19]. Consequently, CS has been extensively exploited in many biomedical applications, including drug, growth factor and gene delivery, tumor targeting, wound dressing, and tissue regeneration [18,19,20,21,22,23,24,25,26]. CS is used as a natural polymeric excipient in many drug delivery dosage forms, including capsules, tablets, films, gels, nanoparticles, microparticles, and transdermal patches [20,21,22,23,24,25,26]. The high content in –NH_2_ and –OH functional groups also makes CS an excellent chelating agent, being involved in the removal of various pollutants, such as heavy metal ions and dyes [27]. On the other hand, CS has also been used in food products since it safeguards the food from pathogen attack [28], enhances the gelation of seafood products, or can be employed in fruit juice deacidification [29,30]. However, CS has some major disadvantages, such as it is soluble only in acidic pH, has a poor barrier to moisture, and exhibits weak mechanical properties. To address these drawbacks, numerous studies have been dedicated to chemical modification of CS by attachment of new functional moieties [31,32] or by cross-linking with di- or polyfunctional agents, such as epichlorhydrin, GA, sodium tripolyphosphate, methoxysalicylaldehyde, and ethyleneglycol diglycidyl ether [21,27,33]. Moreover, to tailor the characteristics of CS, blending or graft copolymerization with other synthetic or natural polymers to form 3D hydrogels with enhanced water retention capacity, without losing their structural integrity, has also been considered [34,35,36].

Lately, porous composite hydrogels [37,38] and shape memory polymers [39] have attracted great interest, since they are characterized by a fast response rate at small modifications of the external stimuli and can afford excellent self-healing, good re-processability, and high mechanical performance. The techniques currently applied to design such materials include directional freeze-drying, ice-templating (cryogelation), freeze-thawing, porogen leaching, gas foaming, electrospinning, 3D printing, colloidal crystal templating, and photolithography [17,18,19,21,22,23,37,38,39]. Among them, cryotropic gelation is one versatile and eco-friendly approach, which meets the requirements of the food and pharmaceutical industries, to design functional hydrogel matrices [40,41,42,43]. A unique feature of cryogelation is represented by the low temperatures at which the reactions are conducted, which are highly advantageous for encapsulating temperature-sensitive bioactive compounds, such as proteins [44]. Hedström, el al. demonstrated the successful encapsulation of an enzyme within albumin/CS-based cryogels as well as the preservation of its bioactivity [44].

Recently, our group reported the preparation of novel composite cryogels based on poly(N,N-dimethylaminoethyl methacrylate) and poly(acrylamide) [45], porous polyelectrolyte complexes (PECs) based on CS and carboxymethylcellulose or poly(2-acrylamido-2-methylpropanesulfonate sodium salt [46], and oxidized starch [47] as suitable drug delivery systems for loading and release of CCM. In the present study, we aim to develop novel polymeric formulations based only on cross-linked CS capable of providing adequate stability and sustained release of CCM. The obtained cross-linked CS sponges were characterized by FTIR spectroscopy, SEM, swelling degree, and uniaxial compression measurements as a function of CS concentration, cross-linking ratio, and gelation conditions. To establish the potential therapeutic functionality of these sponges, the CCM release profile and the antibacterial and antioxidant activities were evaluated. In addition, this work will prove the benefits of cryogelation instead of conventional gelation at room temperature for the preparation of porous CS-based materials.

## 2. Results and Discussion

**Scheme 1:**
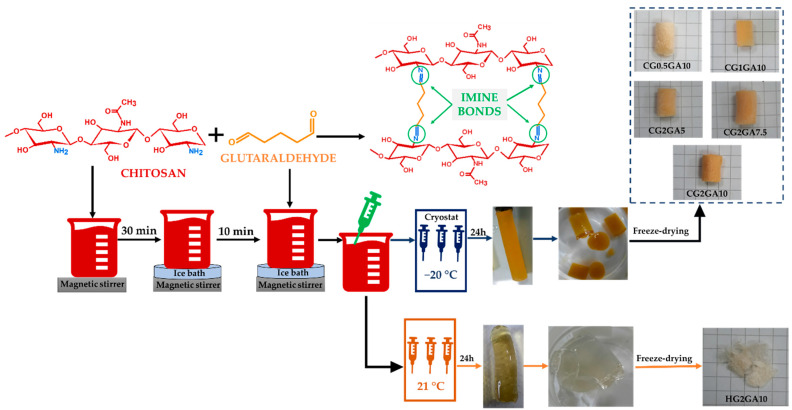
Preparation procedure of CS sponges by cryogelation and by RT gelation.

In this work, GA-cross-linked CS sponges were prepared either by cryogelation (−20 °C) or by room-temperature (RT) gelation (21 °C), according to the protocol depicted in Scheme 1 and presented in Section 3.2. Briefly, CS solutions of different concentrations were first cooled on ice baths, and then GA solutions were added to ensure pre-established ratios between CS and cross-linker (Table 1). Subsequently, the obtained mixtures were drawn into syringes that were kept for 24 h either in a cryostat at −20 °C or at RT (21 °C) to allow the cross-linking reaction to proceed. GA is a well-known cross-linker that reacts with the amino groups of CS, forming imine bonds (Scheme 1) [48,49,50].

The influence of different CS concentrations and preparation conditions on the gel-forming ability and the aspect of sponges are shown in Table 2. As presented, by adding 0.29 μmol of GA, opaque gels were obtained by cryogelation, irrespective of the CS concentration. The opaqueness is an inherent property of cryogels that stems from an extended network of porous inclusions, which scatter light [51]. On the other hand, at RT, gelling occurred only at 2 wt. % CS concentration, the obtained gel being transparent and light yellow. Other CS concentrations did not form gels, probably because there were not sufficient interactions between CS chains and GA molecules to generate stable 3D architectures. From here on, the gels prepared by cryogelation will be described by the general formula CGxGAy, where CG originates from the word cryogels, while x and y indicate the concentration of the stock solutions of CS and, respectively, GA used to prepare the corresponding samples. Further, for the hydrogel prepared at RT using 2 wt. % CS and 10 wt. % GA stock solutions, the term HG2GA10 will be used.

The two preparation strategies also strongly influenced the outer aspect of the obtained sponges (optical images in Scheme 1). Thus, all cryogels exhibited a monolithically aspect, and their colour varied from light yellow to dark brown, depending on the composition (Table 1). The enhanced shape stability of cryogels arises from the cryostructuration procedure, in which the formation and growing of ice crystals lead to the coalescence of all reagents (CS chains and GA molecules) into confined regions, where they react. This induces the formation of tightly bound intertwined polymeric regions (i.e., pore walls) that ultimately translate into a strong and elastic polymeric skeleton [52,53,54]. On the other hand, the HG2GA10 sample swelled excessively in water and lost its monolithic aspect during purification (optical images in Scheme 1).

### 2.1. GFY and Density

*GFY* is a measure of the content of dry cross-linked polymeric networks versus the dry weight of used reagents (Equation (1), Section 3.3.1). As presented in Table 1, the *GFY* of prepared cryogels varied from 86.28 ± 0.65% to 89.37 ± 0.48%, while for the HG2GA10 sponge, a *GFY* of 93.71 ± 2.4% was determined. It can be concluded that most of the used reagents were successfully incorporated into the final sponges.

**Figure 1:**
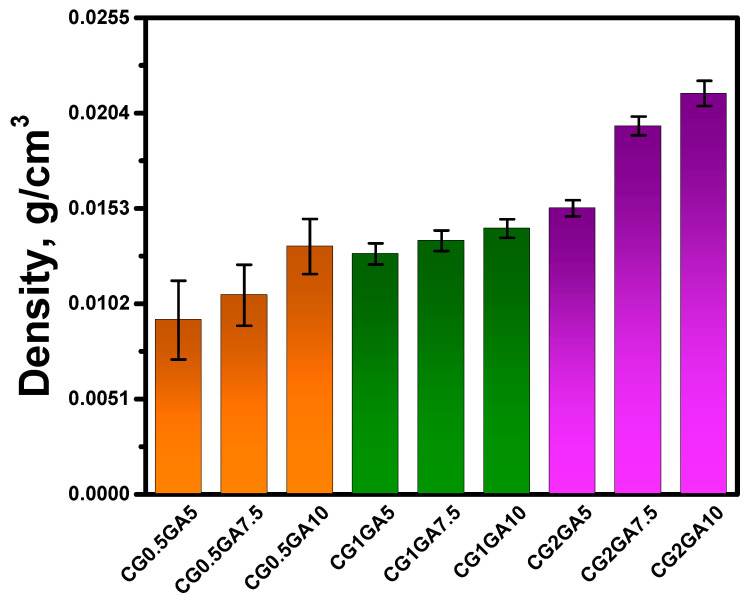
Density of CS cryogel sponges prepared with various initial CS concentrations and cross-linking degrees.

As stated above, cryogels accommodate an extended network of macropores that is obtained after the removal of ice crystals. Consequently, they are usually characterized by low density, which varies with their composition. Hence, the density of all prepared cryogel sponges was evaluated from their weight and volume (Equation (2), Section 3.3.2). As depicted in Figure 1, the cryogels density increased from 0.00932 ± 0.0021 g/cm^3^ to 0.02145 ± 0.00067 g/cm^3^ by increasing both CS and GA concentrations.

The obtained density values are of the same order of magnitude, or even lower, in comparison to other polysaccharide-based sponges reported in the literature. For example, density values of 0.042–0.175 g/cm^3^ have been reported for CS aerogels cross-linked with formaldehyde [55], while the density of CaCl_2_ cross-linked sodium alginate aerogels ranged between 0.023 g/cm^3^ and 0.062 g/cm^3^ [56]. In another work, Tripathy et al. reported densities from 0.0043 g/cm^3^ to 0.1102 g/cm^3^ for cellulose diacetate aerogels, and from 0.0193 g/cm^3^ to 0.0933 g/cm^3^ for cellulose diacetate hydrogels [57]. In conclusion, the very low densities of the CS-GA cryogels prepared herein recommend them as ultra-light-weight materials.

### 2.2. Structural Characterization

**Figure 2:**
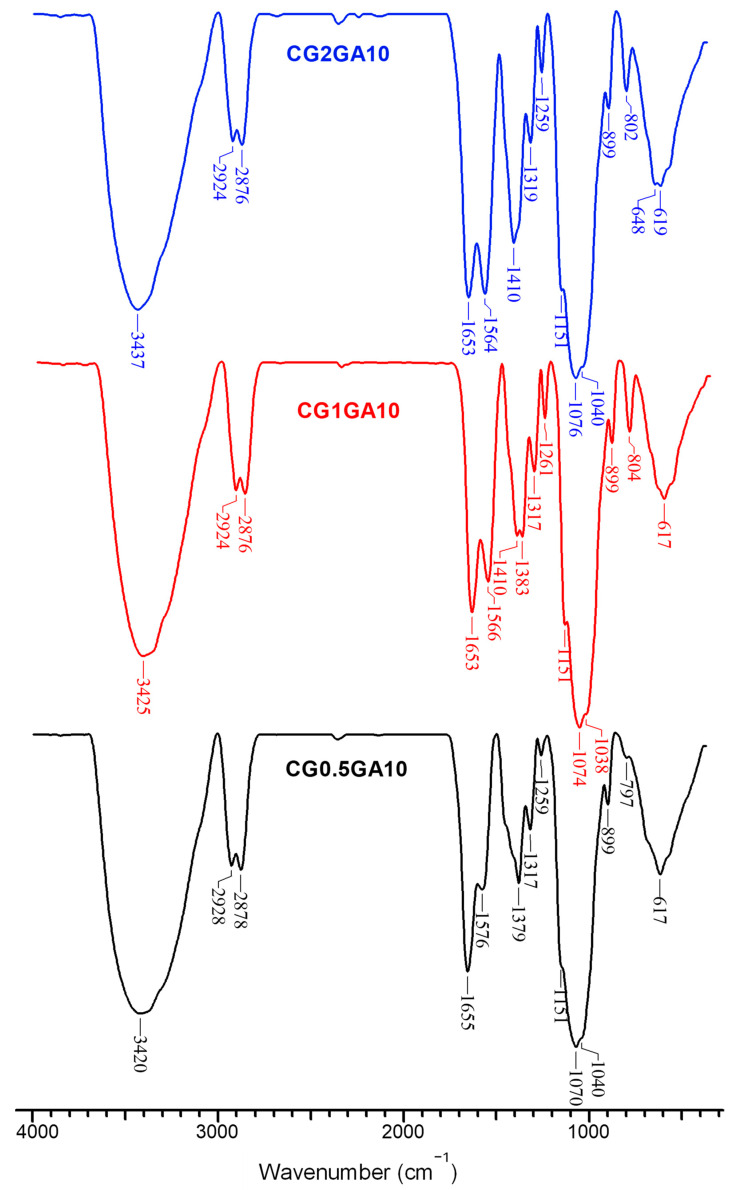
FTIR spectra of CG0.5GA10, CG1GA10, and CG2GA10 cryogels.

The FTIR spectra of all CS sponges prepared in this work are presented in Figure 2 and Figures S1–S3, Supplementary Materials.

The main characteristic bands of GA cross-linked CS sponges can be identified at approximately 3420–3449 cm^−1^, assigned to the –NH and –OH stretching vibrations, as well as to hydrogen bonds [46,47,58,59]; 2922–2928 cm^−1^ and 2874–2878 cm^−1^ attributed to the stretching vibrations of C-H bonds [46,47,58,59]; 1651–1657 cm^−1^ correspond to the stretching vibration of the C=O bonds from the N-acetyl groups (amide I band) [46,47,58,59]; 1564–1593 cm^−1^, assigned to the –NH_2_ bending vibrations; 1410–1412 cm^−1^ attributed to the bending vibrations of –CH_2_ groups; 1379–1387 cm^−1^ correspond to deformation vibrations of –CH_2_ groups. The bands located at 1317–1319 cm^−1^ are assigned to the stretching vibrations of C-N bonds (amide III) [46,47,58,59]. Further, the bands located at 1148–1155 cm^−1^ were attributed to the anti-symmetric stretching vibrations of C−O−C bridges, the bands situated at 1070–1078 cm^−1^ and 1034–1045 cm^−1^, assigned to skeletal C−O bonds stretching vibrations, while the small peak at 897–899 cm^−1^ corresponds to the wagging vibrations of the glycosidic structure [59,60]. Important spectral modifications can be observed for sponges with different CS concentrations but the same GA content: (i) the downshift and steady intensification of the bands corresponding to the bending vibration of –NH_2_ groups that indicates the formation of a higher number of imine cross-links [61] and (ii) the intensification of the bands corresponding to the bending vibrations of –CH_2_ groups (1410–1412 cm^−1^), which could be related to the occurrence of new vibrations from the methylene groups in GA.

### 2.3. Morphology and Elemental Analysis

**Figure 3:**
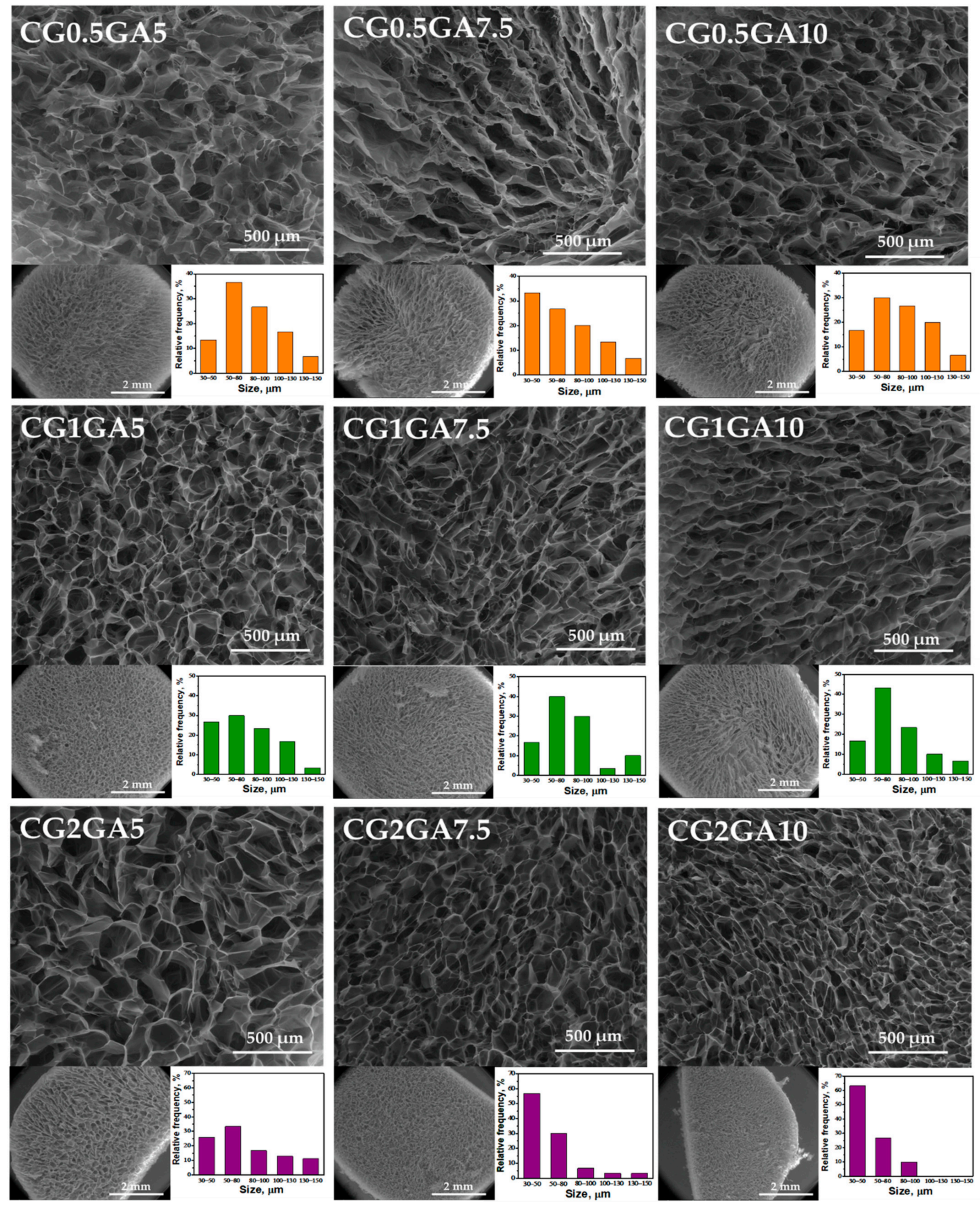
SEM micrographs and pore size distribution of CS sponges prepared by cryogelation (magnification: 150×).

The internal morphology of CS cryogel and hydrogel sponges was investigated via SEM (Figure 3 and Figure S5). As seen in Figure 3, the cryogels exhibited a heterogeneous porous morphology, with interconnected honeycomb-like pores, characteristic for materials prepared via cryogelation [52,53,54,62]. The pore size distribution diagrams depicted in Figure 3 indicate a strong influence of CS and GA concentrations on the cryogels inner morphology, especially for CG2GAy samples. The pore sizes decreased as the cross-linking ratio and the CS concentration increased (CG2GAy samples, Table S1, Supplementary Materials), indicating the formation of more cross-links in the sponges prepared at higher CS and/or GA concentrations (Figure 1). In the case of CG1GAy cryogels, the correlation between the mean pore size diameters and cross-linking degree is not so obvious (Figure 1 and Table S1, Supplementary Materials). Thus, large polydispersity in the pore diameters can be observed in the CG1GA5 sample, whereas by increasing the cross-linking degree (CG1GA7.5 and CG1GA10 samples), the relative frequency (%) of the pores in the range of 50–80 μm increased. By contrast, the CG0.5GAy cryogels did not show such dependence, probably because the concentration of CS is too low, which led to a heterogeneous distribution of the pores interconnectivity along with an enlargement of the pore sizes, demonstrated also by the large error bars of their densities (see Figure 1). On the other hand, the pores of the HG2GA10 sample prepared by RT gelation were less clearly defined (Figure S5, Supplementary Materials), as a result of the statistical gelation in the absence of ice crystal templates. It should be pointed out that a stable porous structure with a relative homogeneous distribution of pore interconnectivity, i.e., about 60% of pores in a range 30–50 μm, can be achieved using 2 wt. % CS and 10 wt. % GA stock solutions (CG2GA10 sample, Figure 1). The EDX elemental analysis (Table S2) evidenced only the presence of C, N, and O atoms, as expected.

### 2.4. Mechanical Properties

**Figure 4:**
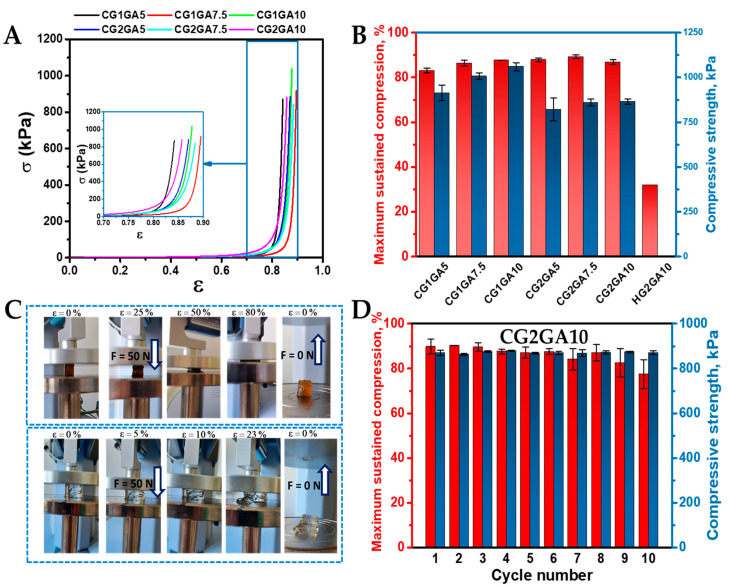
(**A**) Stress–strain profiles of CS sponges. (**B**) Maximum sustained compression (red bars) and compressive strength (blue bars) of CS sponges. (**C**) Optical images of CG2GA10 (upper images) and HG2GA10 (bottom images) sponges under uniaxial compression showing their compression and shape recovery. (**D**) Maximum sustained compression (red bars) and compressive strength (blue bars) of CG2GA10 cryogels under cyclic stress–strain measurements.

The uniaxial compression stress–strain measurements were performed to investigate the mechanical resistance of CS sponges under high-wear stress. Because the cryogels prepared at 0.5 wt. % CS showed poor mechanical features, they were not characterized. Figure 4A depicts the compressive stress–strain (*σ–ε*) profiles of CS cryogels prepared at 1 wt. % and 2 wt. % CS concentration and different cross-linking degrees.

All cryogels were successfully compressed to >80% strain (Figure 4B), without the appearance of deformation or fracture (Figure 4C), this being associated with the complete release of water from their inner structure upon compression. The cryogels prepared at 2 wt. % CS exhibited lower compressive strengths (820.35 ± 63.61 kPa, 860.35 ± 20.31 kPa and 865.96 ± 14.87 kPa for CG2GA5, CG2GA7.5, and CG2GA10 samples, respectively) than the ones prepared at 1 wt. % CS (913.89 ± 43.01 kPa, 1007.32 ± 18.51 kPa and 1060.28 ± 22.97 kPa for CG1GA5, CG1GA7.5, and CG1GA10 samples, respectively). This is correlated with the higher elastic moduli of the sponges prepared at 2 wt. % CS (6.03 ± 1.73 kPa, 11.21 ± 3.49 kPa and 12.53 ± 3.37 kPa for the CG2GA5, CG2GA7.5, and CG2GA10 cryogels, respectively) compared to 1.48 ± 1.01 kPa, 3.96 ± 0.27 and 4.57 ± 0.63 kPa for the CG1GA5, CG1GA7.5, and CG1GA10 samples, respectively (Table S3), which shows that the elasticity of the sponges increased with the increase of CS concentration and of the cross-linking degree. On the other hand, the HG2GA10 sponge withstood a compression of only 32% (Figure 4B) before fracture (Figure 4C).

All cryogels recovered their original shape in a matter of seconds after they were compressed (Video S1 shows the shape recovery of CG2GA10 cryogel, Supplementary Materials), quickly absorbing the surrounding water. This shows that they possess excellent water-triggered shape recovery properties, which stems from their highly porous architecture (Figure 3) that allows for the unhindered and reversible movement of water in and out of their structure. With this in mind, the cyclic stress–strain measurements of CG2GA5 (Figure S6, Supplementary Materials) and CG2GA10 (Figure 4D) were recorded. As seen in Figure 4D, the CG2GA10 cryogel exhibited highly reproducible maximum sustained compression and nominal compression strength values up to ten compression/relaxation cycles. On the other hand, the CG2GA5 sponge preserved their mechanical properties up to only five compression/relaxation cycles (Figure S6, Supplementary Materials), after which cracks were developed and the gel failure occurred. This shows that the shape recovery performance was influenced by the cross-linking ratio, being better as the GA content increased. Therefore, because of their excellent shape recovery properties, the CS cryogels are envisaged as sustainable solutions in various healthcare applications, including drug delivery, wound dressing, or for point-of-demand antimicrobial uses.

### 2.5. Swelling Behavior and Surface Wettability

**Figure 5:**
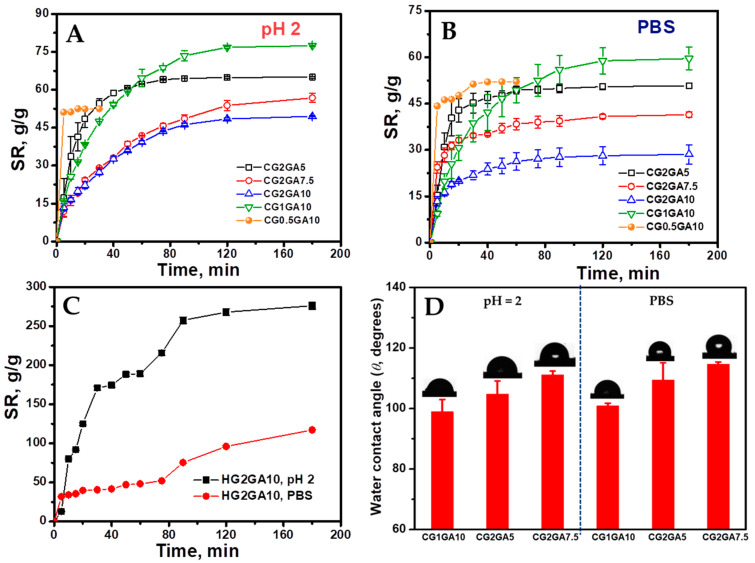
Swelling kinetics of CS cryogels in pH 2 (**A**) and in PBS (**B**) (the CG0.5GA10 cryogel broke at 30 min in pH 2 and at 1 h in PBS); swelling kinetics of CS hydrogel (**C**); water contact angle of CS cryogels (**D**) (the CG2GA10 cryogel was not measured due to its remarkable elasticity; the compressed samples exhibit self-shape recovery).

The water sorption capacity of CS-based cryogels and hydrogels was evaluated in aqueous solutions of pH 2 and phosphate-buffered saline (PBS, pH = 7.4) (Figure 5), since this parameter is associated with ionization of NH_2_ groups, polymer chain relaxation, and dissociation of hydrogen bonds [58]. The values of swelling ratio (*SR*) for all sponges increased in time until the swelling equilibrium was attained. As expected, the highest values of equilibrium *SR* were obtained for the CG1GA10 cryogels, irrespective of the tested medium (Figure 5A,B). The CG0.5GA10 cryogels were not stable and broke at 30 min in pH 2 and at 1 h in PBS. By contrast, the most stable cryogels were those obtained with a CS concentration of 2 wt. %. As can be seen, from Figure 5A,B, the *SR* values increased with a decrease in the cross-linking degree. Thus, the CG2GA10 cryogel reached an *SR* value of 49.48 g/g, whereas an *SR* value of 64.96 g/g was calculated for the CG2GA5 cryogel at pH 2 (Figure 5A). At pH 7.4, the *SR* values were significantly lower (28.59 g/g for CG2GA10 and 50.76 g/g for CG2GA5) (Figure 5B). On the other hand, the CS-based hydrogel prepared at RT (sample HG2GA10) exhibited high values for *SR* but a longer time to attain the equilibrium (Figure 5C). Similar behavior was previously reported for conventional CS-based hydrogels [62,63].

Wettability is an important surface property of hydrogel-based materials since it provides useful insights about the synergies between liquid and solid surface interactions, which are essential in many biological processes [63]. The wetting and un-wetting properties of solid surfaces are generally evaluated by measuring the contact angle (*θ*) of a droplet of water on a solid surface using contact angle goniometer equipment [63,64]. The Young’s equation was applied to calculate *θ* for a liquid on a flat and smooth surface, whereas Wenzel, Cassie and Baxter models were used to describe the relation between apparent and intrinsic contact angles of a droplet on a rough surface [63,64]. It was established that the values of *θ* are affected by several factors, such as drop size, surface roughness, pore structure, substrate absorption rate, and surface tension [63,64,65]. Thus, when *θ* < 90°, the surface is hydrophilic; when *θ* > 90°, the surface becomes hydrophobic, whereas when *θ* > 150°, the surface is super-hydrophobic [63,64,65]. In this work, the surface wettability of CG1GA10, CG2GA5, and CG2GA7.5 cryogels, as films, was evaluated by measuring the contact angle of water droplets with pH 2 or PBS (pH = 7.4) (Figure 5D). CS-based films with a smooth and flat surface (see optical images and SEM micrographs, Figure S7, Supplementary Materials) were obtained by applying a compressive force of 450 N at a rate of 1 mm/min to freeze-dried samples using the Shimadzu Testing Machine. The increment in contact angle with an increase in CS concentration (98.94 ± 4° for CG1GA10, 104.77 ± 4° for CG2GA5, at pH 2, and 100.86 ± 1° for CG1GA10 and 109.47 ± 6° for CG2GA5 at pH 7.4) and cross-linking degree (111.11 ± 1° for CG2GA7.5, at pH 2 and respectively 114.7 ± 1° at pH 7.4) point out the rise in cryogel hydrophobicity (Video S2 indicates the surface hydrophobicity of CG2GA7.5 cryogel) and sustain the swelling ratio results. These data are also well-correlated with already reported wettability data on polysaccharide-based materials [50,58].

### 2.6. Antimicrobial Activity

**Figure 6:**
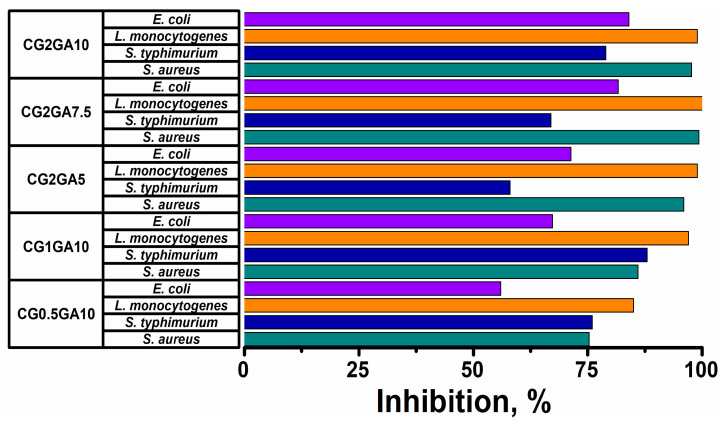
Bacterial inhibition performance of cryogels with varying CS content and cross-linking degree.

CS and CS-based materials have long been under scrutiny for their excellent antimicrobial properties [47,66,67,68,69]. Hence, the antibacterial activity of CS sponges with different CS concentrations and cross-linking degrees has been evaluated against two Gram-negative (*E. coli*, *S. typhymurium*) and two Gram-positive (*L. monocytogenes*, *S. aureus*) bacterial strains (Figure 6).

As seen in Figure 6, the CS sponges more efficiently inhibited the growth of Gram-positive bacteria, but they were less efficient against Gram-negative strains. The composition of the sponges (CS concentration and cross-linking degree) strongly influenced their antibacterial performance. The highest bacterial inhibition was recorded against *L. monocytogenes* and *S. aureus*, whose inhibition increased from 85% up to 100%, and from 75.33% to 99.33%, respectively, by increasing the CS concentration in the sponges from 0.5 wt. % to 2 wt. %. The cross-linking degree in the cryogels strongly influenced their antibacterial performance against the Gram-negative strains (*E. coli* and *S. typhimurium*). Thus, for the cryogels prepared at 2 wt. % CS, the inhibitory performance against *E. coli* and *S. typhimurium* increased from 71.33% to 84 %, and from 58% to 79%, respectively, by increasing the GA cross-linking from 5% to 10% (Figure 6). These findings may be attributed to the pronounced hydrophobicity of the cryogels (low water swelling and high contact angle values—Figure 5, Video S2 indicates the surface hydrophobicity of CG2GA7.5 cryogel).

The antibacterial activity of CS is also associated with its ionic and physical properties, which are significantly influenced by the mode of cross-linking [70]. In the case of Gram-negative bacteria, there are two possible mechanisms of action. One is related to the chelation of various cations, important for the bacterial cell (Ca, Mg) when the pH is above pKa, and the other is the electrostatic interaction of CS with the anionic parts of lipopolysaccharides on the outer membrane. Some studies claim that CS is even able to penetrate the cell membrane in Gram-negative bacteria and interfere with DNA/RNA synthesis [71]. On the other hand, in the case of Gram-positive bacteria, it was shown that CS binds non-covalently to teichoic acid embedded in the peptidoglycan layer, which leads to disruption in cell functions and cell death [72].

### 2.7. CCM-Loaded CS Sponges

CCM (Figure S8A) was loaded into the CS sponges via adsorption from an ethanolic solution, according to the methodology described in Section 3.4. The orange color of all sponges clearly supports the CCM loading into the CS sponges (Figure S8B). The loading efficiency (*LE*, %) and drug loading capacity (*DL*, %) values, calculated according to Equations (5) and (6), are listed in Table S4.

**Figure 7:**
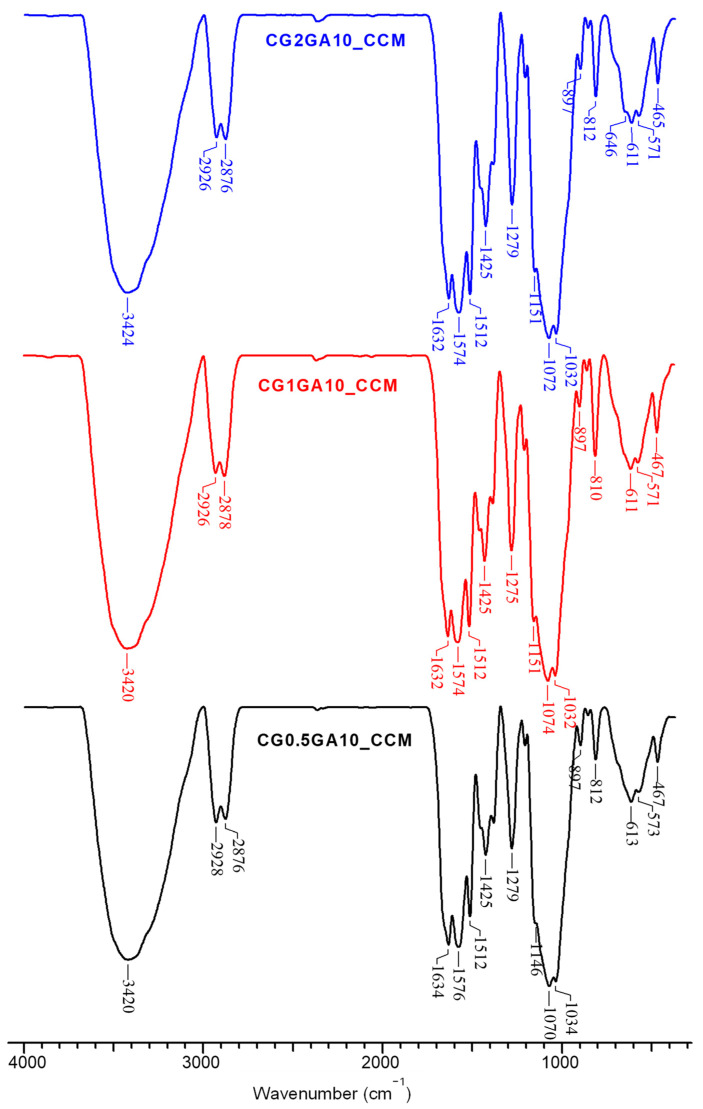
FTIR spectra of CG0.5GA10, CG1GA10, and CG2GA10 cryogels loaded with CCM.

Information about the functional groups of CS sponges after loading of CCM was acquired by FTIR spectroscopy (Figure 7, Figures S3 and S4, Supplementary Materials).

Compared to the FTIR spectra of initial CS sponges (Figure 2 and Figures S1–S3, Supplementary Materials), multiple spectral differences were visible in the FTIR spectra of all sponges loaded with CCM. Thus, the new bands located at 1510–1512 cm^−1^, 1275–1279 cm^−1^, and 810–812 cm^−1^ are associated with the stretching vibrations of aromatic C=C bonds, enolic C–O stretching vibrations, and aromatic C-H vibrations in CCM, respectively [7,73,74]. Furthermore, the blue shift of the bands corresponding to –NH and –OH stretching vibrations, as well as of the amide I bands in CS, clearly supports the interaction between the ketone groups of CCM and the functional groups of CS, probably by hydrogen bonds [74]. Moreover, the slight red shift of the bands associated with the bending vibrations of –CH_2_ groups could also be attributed to the incorporation of CCM into the CS sponges.

**Figure 8:**
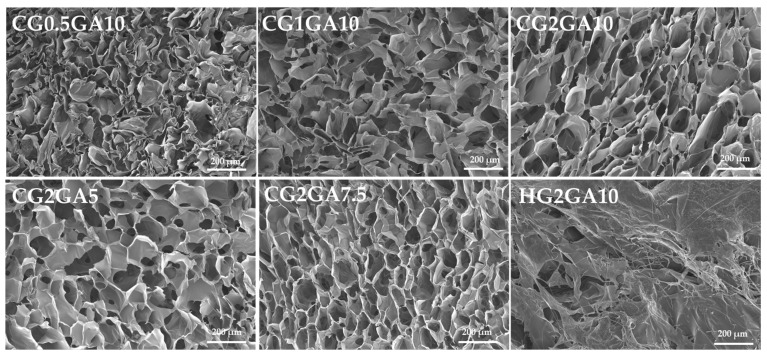
SEM micrographs of CS sponges after loading of CCM (magnification: 100×).

The SEM micrographs of CCM-loaded CS sponges are presented in Figure 8.

As seen in Figure 8, some morphological modifications can be observed in the case of CS cryogels, such as a decrease in pores sizes and even their partial collapse (as noted for the CG0.5GA10 sample), which could be the result of the ethanolic-induced contraction of CS chains during the CCM loading step. The pore sizes of CCM-loaded CS sponges, evaluated using ImageJ software, are presented in Table S1, Supplementary Materials. The highest decline in the mean pore sizes was recorded for the CG0.5GA10 sample (from 82.82 µm to 43.91 µm), followed by the CG2GA5 (from 83.96 µm to 51.24 µm) and CG1GA10 (from 76.54 µm to 60.52) cryogels. On the other hand, the CG2GA7.5 and CG2GA10 cryogels did not exhibit any decrease in the mean pore sizes (Table S1, Supplementary Materials), as a result of their excellent mechanical properties. Consequently, it is obvious that the contraction of CS cryogels strongly depended on their composition (CS concentration and cross-linking degree). By contrast, the HG2GA10 hydrogel preserved its original smooth morphology.

The loading of CCM into the CS sponges was also supported by the EDX elemental analysis, by the increase in oxygen percentage into the CCM-loaded samples compared to the pristine ones (Table S2, Supplementary Materials).

### 2.8. In Vitro Release of CCM

**Figure 9:**
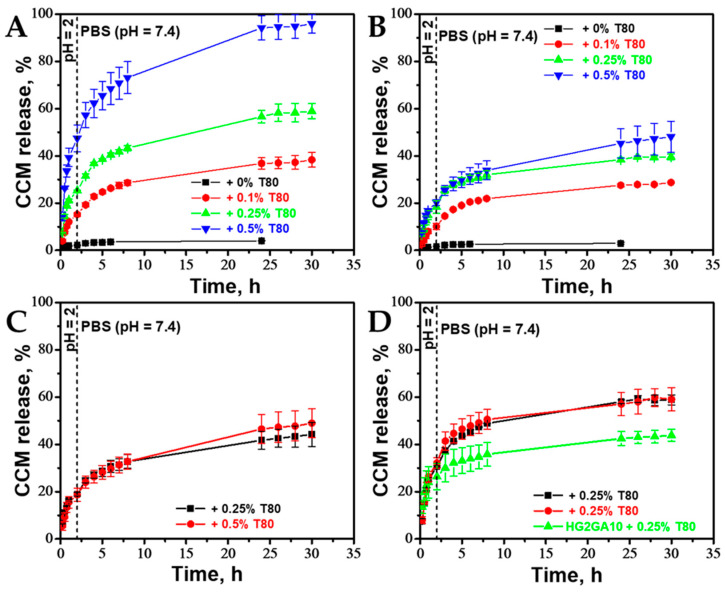
Release kinetics of CCM (at 37 °C in pH 2 and PBS solutions) from (**A**) CG2GA5 sponges with different T80 concentrations, (**B**) CG2GA10 sponges with different T80 concentrations, (**C**) CG2GA7.5 cryogels with different T80 concentrations, and (**D**) CG0.5GA10, CG1GA10, and HG2GA10 sponges at 0.25 wt. % T80.

The CCM release kinetics were investigated in simulated gastrointestinal media, first at pH 2 for 2 h, then changing the release medium to PBS (Figure 9). Because CCM is a poorly water-soluble drug, but is more soluble in lipidic media, we also investigated the influence of Tween80 (T80), a common food additive, on CCM release profiles. T80 (or polysorbate 80) is a non-ionic surfactant that interacts with CCM via hydrophobic interactions, increasing its solubility and improving its bioavailability [8,75].

As displayed in Figure 9A,B, the CCM release from the CG2GA5 and CG2GA10 cryogels was very low in the absence of T80. However, the release kinetics of CCM were dependent on the concentration of T80, being faster as the concentration of T80 increased (Figure 9A–C). The CCM release kinetics was also influenced to a large extent by the composition of CS sponges, i.e., CS concentration and cross-linking degree. Thus, the CCM release was faster in the case of CG2GA5 sponges (Figure 9A) compared with CG2GA7.5 (Figure 9C) and CG2GA10 (Figure 9B) sponges, irrespective of T80 concentration. For example, at 0.5 wt. % T80 in the release medium, the CCM release at 30 h reached 95.91 ± 3.97%, 48.94 ± 6.08%, and 48.09 ± 6.64% for the CG2GA5, CG2GA7.5, and CG2GA10 cryogels, respectively. Concerning samples with the same cross-linking degrees, but prepared at different CS concentrations, the CCM release kinetics from the CG0.5GA10 and CG1GA10 cryogels were similar (Figure 9D), but they were much slower for the CG2GA10 sample (Figure 9B). Thus, the cumulative release of CCM at 30 h in media containing 0.25% T80 reached 58.82 ± 2.12%, 59.05 ± 4.94%, and 39.56 ± 1.16% for the CG0.5GA10, CG1GA10, and CG2GA10 sponges, respectively. This behavior could be correlated with the mean pore sizes of the sponges in aqueous media (Table S1 lists the mean pore sizes of the sponges after drying from water) that were much higher for the CG0.5GA10 and CG1GA10 cryogels than for the CG2GA10 sponge. Lastly, slightly steeper release kinetics were found for the HG2GA10 hydrogel (Figure 9D) compared to the CG2GA10 sponge (Figure 9B) in media containing 0.25% T80, the cumulative release values recorded at 30 h being 43.84 ± 2.53% and 39.56 ± 1.16%, respectively. Overall, the presented data provide important insight into the possibility to modulate the release of CCM, a poorly water-soluble polyphenol, from CS sponges by changing either the release medium or their composition and preparation pathway.

**Figure 10:**
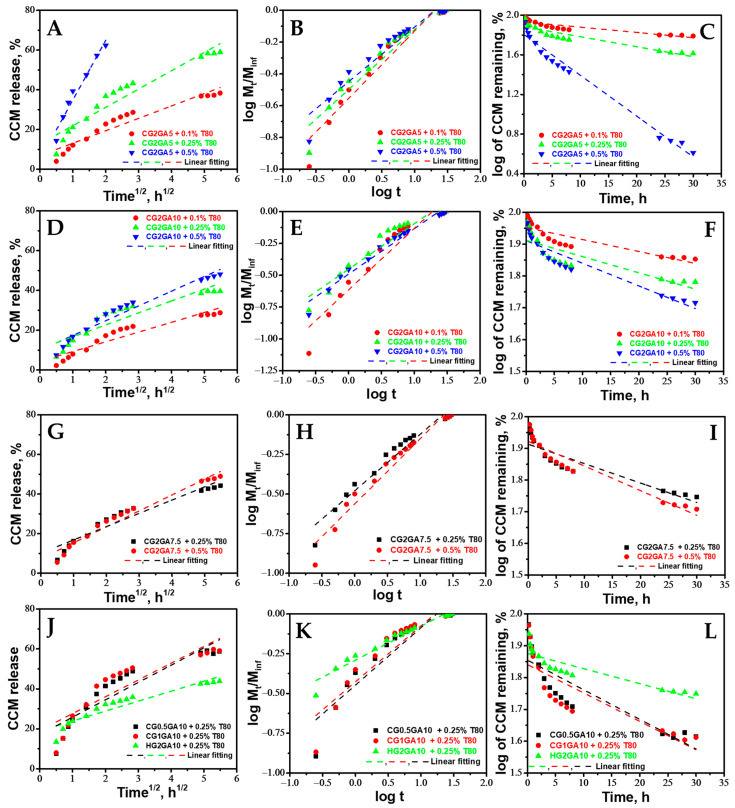
The Higuchi (**A**,**D**,**G**,**J**), Korsmeyer–Peppas (**B**,**E**,**H**,**K**), and first-order kinetic model (**C**,**F**,**I**,**L**) fitting profiles of CCM release data from CG2GA5 sponges with different T80 concentrations (**A**–**C**), CG2GA10 sponges with different T80 concentrations (**D**–**F**), CG2GA7.5 cryogels with different T80 concentrations (**G**–**I**), and CG0.5GA10, CG1GA10, and HG2GA10 sponges at 0.25 wt. % T80 (**J**–**L**).

Information on the release kinetics mechanism of CCM from the CS sponges was obtained by fitting the experimental data with the Higuchi (Equation (7)), Korsmeyer–Peppas (Equation (8)), and first-order (Equation (9)) kinetic models. The fitting results are presented in Figure 10, and the obtained kinetic parameters and *R*^2^ values are listed in Table 3.

From the coefficients of determination, *R*^2^, presented in Table 3, it is seen that the CCM release kinetics were fitted better by the Korsmeyer–Peppas for all CS sponges (*R*^2^ = 0.8585–0.9708), irrespective of the tested release media, with the exception of the CG2GA5 sample in the 0.5% T80 medium, for which the release kinetics were fitted best by the first-order kinetic model (*R*^2^ = 0.9813). The obtained fitting result for the CG2GA5 cryogel indicates that the CCM release is concentration-dependent [76], as a result of its very good solubility in 0.5% T80 aqueous solutions and high porosity of this sponge. The release mechanism of CCM could be projected based on the values of the diffusional exponent (*n_r_*) in the Korsmeyer–Peppas model. As seen in Table 3, the values of *n_r_* were lower than 0.5 for all sponges and T80 concentrations, indicating pseudo-Fickian diffusion release mechanism [47].

### 2.9. Antioxidant Activity

**Figure 11:**
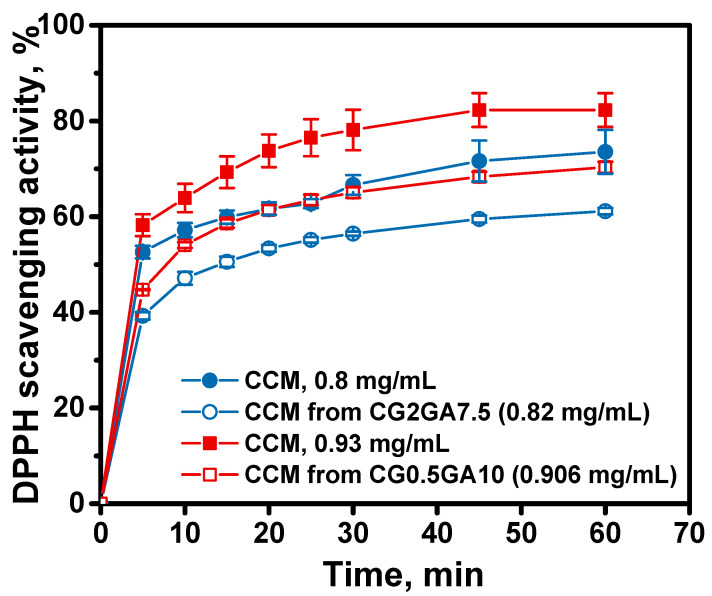
DPPH radical scavenging kinetics of pure CCM and CCM extracted from CS cryogels.

The antioxidant activity of CCM is attributed to its capacity to remove reactive oxygen and nitrogen, to chelate metal ions, and to regulate the function of many enzymes [77,78]. Hence, in preliminary experiments, we investigated the radical scavenging performance of CCM extracted from CG0.5GA10 and CG2GA7.5 sponges, as a function of time, by the 2,2-diphenyl-1-picrylhydrazyl (DPPH) method (Figure 11). For control, free CCM was also tested. According to the literature, the antioxidant compounds quench the single electron of DPPH radical (violet color), converting it to non-radical form (yellow color) [58,79]. This process is accompanied by a decrease in DPPH radical’s characteristic absorbance at 517 nm.

As Figure 11 shows, both free CCM and the CCM extracted from the CS sponges presented a time-dependent radical scavenging activity. The DPPH quenching was very fast in the first 5 min and then steadily equilibrated up until 1 h. An approximately 15% decline in CCM’s radical scavenging activity was determined after incorporation into the CS sponges. However, the antioxidant activity of CCM extracted from the CS cryogels is still high enough, indicating that CCM is protected from degradation by encapsulation into the cryogels.

## 3. Materials and Methods

### 3.1. Materials

Chitosan (CS), GA aqueous solution (25%, *w*/*w*), CCM (96%), PBS, DPPH, and T80 were purchased from Sigma-Aldrich (Steinheim, Germany) and used as received. Glacial acetic acid, methanol, ethanol, and HCl (37%, *v*/*v*) were purchased from Chemical Company (Iasi, Romania). Millipore-grade water was used throughout all experiments. The average viscosity molecular weight and the deacetylation degree of CS, determined according to previously described methodologies [80], were 189 kDa and 85%, respectively.

### 3.2. Preparation of CS Sponges

CS sponges were prepared by either cryogelation or by RT gelation at different CS concentrations and/or GA cross-linking degrees by the following protocol. First, a 2 wt. % CS stock solution was prepared in a 2 wt. % aqueous acetic acid solution. GA stock solutions at concentrations of 5, 7.5, and 10 wt. % were also prepared by appropriate dilutions of purchased GA. CS solutions of different concentrations (see Table 2) were prepared from the stock solution by appropriate dilutions and were homogenized by stirring for 30 min in a magnetic stirrer. To illustrate the synthetic strategy, we present details for the preparation of CG2GA10 cryogels. Typically, 0.28 mL aqueous solution of GA, with a concentration of 10 wt. %, was added drop by drop under vigorous magnetic stirring in 10 g aqueous solution of 2 wt. % CS. The obtained mixture was cooled and stirred in an ice bath for 1 h. Then, the reaction mixture was transferred into 2 mL plastic syringes. The plastic syringes were used as containers since they are an excellent choice for preparing regular monolith-shaped samples. The syringes containing precursor solutions were either placed in a cryostat at −20 °C, or were kept at RT (21 °C). After 24 h, they were taken out and were thawed at RT for 1 h. After that, the samples from the syringes were easily pulled out using the piston of the syringe and cut into specimens of approximately 4 mm in length, and they were abundantly washed with a large excess of distilled water to remove the unreacted polymer and cross-linker. The washing water was exchanged every 2 h within the first 10 h, and then every 12 h for 1 week. Finally, the samples were dried via lyophilization in a Martin Christ ALPHA 1–2LD device for 48 h, at −50 °C and 0.05 mbars. The lyophilized sponges were further dried to constant weight under vacuum in the presence of P_2_O_5_. The sponges were prepared in duplicate for GFY evaluation.

### 3.3. Characterization Methods

#### 3.3.1. *GFY*

The conversion of reactants into the CS sponges was evaluated by determining the *GFY*, according to Equation (1):

(1) $$GFY\;\left(\%\right)=\frac{W_{d}}{W_{m}}\times 100$$

where *W_d_* is the weight of dried CS sponges (under vacuum in the presence of P_2_O_5_ until constant weight) and *W_m_* represents the weight of all used reactants [58]. The *GFY* was calculated as average ± standard deviation from two different synthesis batches.

#### 3.3.2. Density

To evaluate the density of CS cryogels in dry state, the volume of prepared monoliths was first determined by measuring their diameter and height using a digital caliper. The volume was calculated using Equation (2), which describes the volume of a cylinder:

(2) $$V=\pi \times r^{2}\times h$$

where *π* is 3.14, *r* (cm) is the monolith radius and *h* (cm) is the monolith height.

Then, the density of CS sponges was determined using Equation (3) [81]:

(3) $$\rho =\frac{m}{V}$$

where *m* (g) is the monolith weight and *V* (cm^3^) is the determined volume. The density values for CS-based cryogels were calculated as average ± standard deviation from at least three different monoliths. For the hydrogel sample, the density could not be determined because it did not preserve the monolith shape after the purification steps.

#### 3.3.3. FTIR Spectroscopy

The FTIR spectra of CS sponges, before and after loading of CCM, were recorded with a Bruker Vertex 70 FTIR spectrophotometer (Bruker, Ettlingen, Germany) in a range 4000–400 cm^−1^, at a resolution of 2 cm^−1^, by the KBr pellet technique. Prior to the analysis, the sponges were first grounded under liquid nitrogen and dried under vacuum in the presence of P_2_O_5_.

#### 3.3.4. SEM, EDX, and Pore Size Analysis

The internal morphology of pristine CS sponges was investigated with a Quanta 200 ESEM microscope (FEI Company, Hillsboro, OR, USA), operating in low vacuum mode, at 20 kV, with secondary electrons. The morphology of CS sponges loaded with CCM was investigated with a Verios G4 UC (Thermo Scientific, Brno, Czech Republic) scanning electron microscope operating in high vacuum mode using an Everhart-Thornley Detector (Thermo Scientific, Brno, Czech Republic) at an accelerating voltage of 5 kV. The elemental composition of sponges was evaluated with an energy-dispersive X-ray (EDX) analyzer (Octane Elect Super SDD detector, Ametek, Berwyn, PA, USA). The pore diameter of CS sponges was evaluated from the SEM micrographs using Image J 1.53 v open-source software, by measuring at least 30 pores (voids) for each sample. The pore size distribution was plotted as a function of the percentage frequency (%) [58].

#### 3.3.5. Mechanical Properties

Uniaxial compression tests were performed at RT on water-swollen sponges (monoliths of 10 mm diameter and 8 mm height) using a Shimadzu Testing Machine (EZ-LX/EZ-SX Series, Kyoto, Japan), at a compression rate of 1 mm/min and an applied force of 50 N. The compressive stress (*σ*) and the strain (*ε*) were calculated according to a previously described methodology [49]. The maximum sustained compression (%), compressive strength (kPa), and elastic moduli (kPa) were calculated as average ± standard deviation from two measurements.

#### 3.3.6. Swelling Properties

The swelling kinetics of CS sponges was evaluated by immersing about 10 mg of dried samples in pH 2 or in PBS (pH 7.4) aqueous solutions. The swollen samples were periodically removed from water and weighed, after wiping the excess of solvent using filter paper. The *SR* (g/g) was calculated according to Equation (4):

(4) $$SR=\frac{W_{t}}{W_{d}}$$

where *W_d_* (g) is the weight of the dried sponges, while *W_t_* (g) is the weight of the water-swollen sponges at time *t*. The swelling measurements were performed in duplicate and the results were expressed as average values ± standard deviation.

#### 3.3.7. Contact Angle Measurements

The static contact angle (*θ*) measurements were performed by the drop method [58,82] for all samples. Briefly, 1 μL of MilliQ water was placed on the surface of each sample, using a CAM-200 instrument. To obtain perfectly flat and smooth films of about 0.1 mm thickness, the freeze-dried CS-based cryogels were compressed using a Shimadzu Testing Machine (EZ-LX/EZ-SX Series, Kyoto, Japan). In this regard, an applied force of 450 N at a compression rate of 1 mm/min was used. The *θ* values were obtained by fitting the Young–Laplace equation onto the drop profile of each sample, and they were calculated as an average of five consecutive measurements.

### 3.4. Loading and Release of CCM

CCM was loaded into the CS sponges by sorption from a ≈5 mg/mL ethanolic solution, up to maximum sorption capacity. Afterwards, they were first kept 2 h in closed vials at 4 °C in the dark. Then, the vials were opened and the CCM-loaded sponges were dried for about 48 h in a vacuum oven at RT in the dark. The loading efficiency of CCM into the CS sponges was evaluated by UV-vis spectrophotometry (SPECORD 200 Analytik Jena), based on a pre-recorded calibration curve for CCM at 431 nm. The residual CCM amount in the glass vials was dissolved in 10 mL of ethanol, and then the CCM concentration in the obtained solution was determined by UV-vis. The loading efficiency (*LE*, %) was calculated based on Equation (5):

(5) $$LE,\;\%=\frac{\left(V_{CCM}\times C_{0}\right)-q_{r}}{V_{CCM}\times C_{0}}\times 100$$

where *V_CCM_* (mg/L) is the volume of CCM solution with a concentration of 5 mg/mL (*C*_0_) and *q_r_* (mg) is the residual amount of CCM remaining in the glass vial.

The drug loading capacity (*DL*, %) was calculated with Equation (6):

(6) $$DL,\;\%=\frac{\left(V_{CCM}\times C_{0}\right)-q_{r}}{m}\times 100$$

where *V_CCM_*, *C*_0_, and *q_r_* have the same meaning as above, and *m* is the weight of the sponge.

The in vitro release of CCM from the CS sponges was evaluated in simulated gastrointestinal fluids, according to a protocol previously published [46,47,83]. Thus, the CCM-loaded CS sponges were immersed first for 2 h in 10 mL of release medium at pH 2 (with or without T80). At predetermined time intervals, 1 mL of the release medium was withdrawn to spectroscopically evaluate the release of CCM, being replaced by 1 mL of fresh release medium to keep the volume constant. After 2 h, the release medium was replaced with 10 mL of PBS (with or without T80), periodically evaluating the release of CCM from the sponges. The cumulative release of CCM was calculated with Equation (7):

(7) $$CCM\;\left(\%\right)=\left[\frac{10C_{n}+\sum C_{n-1}}{m}\right]$$

where *C_n_* and *C_n_*_−1_ are the concentrations of CCM (mg/L) in the releasing medium after *n* and *n-1* withdrawing steps, and *m* is the amount of CCM in the sample [35].

To obtain information on the release mechanism, the CCM release data were fitted with the Higuchi model (Equation (8)) [84], Korsmeyer–Peppas model (Equation (9)) [85], and first-order kinetics model (Equation (10)) [47]:

(8) Mt=kKt12

(9) $$\frac{M_{t}}{M_{\infty }\;}=k_{KP\;}t^{n_{r}}$$

(10) $$M_{t}=M_{0}-exp^{-k_{1}t}$$

where *k_H_* is the Higuchi constant, *M_t_* and *M_∞_* are the cumulative amounts of CCM released at time *t* and the maximum released amount, *k_KP_* is the Korsmeyer–Peppas model constant, *n_r_* is a diffusional exponent that gives information about the release mechanism, *k*_1_ is the constant for first-order model, and *Mo* is the initial amount of drug.

### 3.5. Antimicrobial Activity

The antimicrobial activity of CS sponges was evaluated against two Gram-positive ( *L. monocytogenes*, ATCC 7644 and *S. aureus,* ATCC 25923) and two Gram-negative (*E. coli,* ATCC 25922 and *S. typhymurium*, ATCC 14028) bacteria. The antibacterial tests were carried out in agreement with the International Standard ISO 11133 (guide for the preparation and production of culture media) and SR ISO 7218 (General Directive for microbiological examinations), as previously described [47,86].

The CS sponges were first sterilized (20 min at 110 °C and 0.5 bar); then, they were mixed with the microbial inoculum (300 CFU/g for *E. coli* and *S. aureus*; 100 CFU/g for *S. typhymurium* and *L. monocytogenes*). After incubation for 24 h at 37 °C, 100 µL of the obtained mixtures was seeded on the surface of corresponding medium (XLD—*S. typhymurium*, VRBG—*E. coli*, ALOA—*L. monocytogenes*, Baird Parker—*S. aureus*) and the colonies were counted against a blank (filter paper prepared under the same conditions).

### 3.6. Antioxidant Activity

The radical scavenging activity of CCM-loaded CS cryogels was evaluated by the DPPH assay, according to a previously published protocol, with slight modifications [58]. Briefly, about 40 mg of CCM-loaded samples was grounded and then introduced for 1 h into a volume of extraction medium (1% CH_3_COOH into CH_3_OH solution), calculated to obtain ≈1 mg/mL CCM solution in the extract. Then, the supernatant was separated by centrifugation (10 min at 10,000 rpm) and 50 µL of the extract was mixed with 2.95 mL DPPH solution to determine the radical scavenging activity. The absorbance was measured at 517 nm at pre-established time intervals, under dark conditions. The DPPH radical scavenging activity (%) was calculated with Equation (11):

(11) $$DPPH\;scavenging\;activity,\;\%=\frac{A_{C}-A_{S}}{A_{C}}\times 100$$

where *A_C_* is the absorbance of control samples (mixture of 50 µL methanol and 2.95 mL DPPH solution) and *A_S_* is the absorbance of the sample. The experiments were performed in duplicate and the results are expressed as average values ± standard deviation.

## 4. Conclusions

In this work, new CS sponges with varying composition and complementary characteristics were prepared by cryogelation or by room-temperature gelation, through GA cross-linking. The sponges exhibited honeycomb morphology, excellent mechanical properties, and water-triggered shape recovery, modulated by the concentration of CS, the cross-linking ratio, and gelation conditions. They also showed remarkable antibacterial properties against Gram-positive (*S. aureus*, *L. monocytogenes*) and Gram-negative (*E. coli*, *S. typhimurium*) strains. The investigation of the release properties of CCM, a valuable plant-derived polyphenol, indicated that CCM release was dependent on the sponge composition and preparation strategy. The release mechanism of CCM was estimated by linearly fitting the Higuchi, Korsmeyer–Peppas, and the first-order kinetic models onto the experimental data. Considering the values of the diffusional exponent *n_r_* in the Korsmeyer–Peppas equation, a pseudo-Fickian diffusion-controlled mechanism was identified for the release of CCM. Moreover, the high antioxidant activity values for CCM extracted from the CS cryogels indicate that it is protected from degradation by encapsulation into the cryogels.

## Figures and Tables

**Table 1:** Preparation conditions and GFY (%) of CS sponges.

^a^ Sample Code	CS, wt. %	^b^ GA, wt. %	^c^ GA, μmol	^d^ T, °C	^e^ *GFY*, %
CG0.5GA5	0.5	5	0.14	−20 ± 2	86.28 ± 0.65
CG0.5GA7.5	0.5	7.5	0.22	−20 ± 2	88.96 ± 0.83
CG0.5GA10	0.5	10	0.29	−20 ± 2	89.37 ± 0.48
CG1GA5	1	5	0.14	−20 ± 2	86.28 ± 0.80
CG1GA7.5	1	7.5	0.22	−20 ± 2	86.07 ± 1.11
CG1GA10	1	10	0.29	−20 ± 2	87.47 ± 0.46
CG2GA5	2	5	0.14	−20 ± 2	86.60 ± 0.42
CG2GA7.5	2	7.5	0.22	−20 ± 2	86.60 ± 0.01
CG2GA10	2	10	0.29	−20 ± 2	86.64 ± 0.52
HG2GA10	2	10	0.29	21 ± 2	93.71 ± 2.4

^a^ CG and HG denote samples prepared by cryogelation and by RT gelation, respectively; ^b^ Represents the concentration of the GA stock solutions; ^c^ The amount of GA in μmol added in the reaction mixture calculated to one mmol of NH_2_; ^d^ The gelation temperature; ^e^ Gel fraction yield (*GFY*), determined according to the methodology presented in Section 3.3.1.

**Table 2:** The appearance in wet state of CS sponges prepared at various initial CS concentrations and temperatures.

	^a^ C_CS_	0.5 wt. %	1 wt. %	2 wt. %
T °C				
−20 °C		Opaque; Light yellow	Opaque; Light yellow	Opaque; Dark brown
21 °C		No gel	No gel	Transparent; Light yellow

^a^ Sponges prepared with 0.29 μmol of GA.

**Table 3:** Parameters obtained by linear fitting of CCM release by Higuchi, Korsmeyer–Peppas, and first-order kinetic models.

Sample	wt. % T80	Higuchi		Korsmeyer-Peppas			First Order	
		*k_H_*	*R* ^2^	*n_r_*	*k_KP_* (min^−nr^)	*R* ^2^	*k*_1_	*R* ^2^
CG0.5GA10	0.25	8.6678	0.8392	0.3607	6.3913	0.8807	−0.0093	0.7682
CG1GA10	0.25	8.3991	0.7875	0.3511	6.5265	0.8585	−0.0089	0.7073
CG2GA5	0.1	6.2567	0.9168	0.4236	5.7488	0.9386	−0.0053	0.8225
	0.25	9.2292	0.9264	0.3766	6.0836	0.9395	−0.0101	0.8774
	0.5	30.0719	0.9629	0.3428	6.3910	0.9292	−0.0412	0.9813
CG2GA7.5	0.25	6.7136	0.9236	0.3571	6.1903	0.9542	−0.0061	0.8432
CG2GA10	0.1	4.9026	0.8915	0.4841	5.4034	0.9201	−0.0038	0.7731
	0.25	5.9882	0.8522	0.3583	6.3565	0.9264	−0.0051	0.7387
	0.5	7.4126	0.9498	0.3583	6.1163	0.9708	−0.0071	0.8869
HG2GA10	0.25	4.9796	0.8904	0.2133	7.4776	0.9508	−0.0047	0.7983

## Data Availability

Not applicable.

## Supplementary Materials

The following supporting information can be downloaded at: https://www.mdpi.com/article/10.3390/ijms24054452/s1.
