# Supplementary material for: Chitosan Sponges with Instantaneous Shape Recovery and Multistrain Antibacterial Activity for Controlled Release of Plant-Derived Polyphenols
Source: Int J Mol Sci. 2023 Feb 23;24(5):4452. doi: 10.3390/ijms24054452 (PMC10002852; doi:10.3390/ijms24054452)
Supplement: Supplementary file 1 [file ijms-24-04452-s001.zip › IJMS2023_SI.final.pdf]

SUPPLEMENTARY MATERIAL

Chitosan Sponges with Instantaneous Shape Recovery and Multistrain Antibacterial Activity for Controlled Release of Plant-Derived Polyphenols

Ioana-Victoria Platon, Claudiu-Augustin Ghiorghita, Maria-Marinela Lazar, Irina Elena Raschip, Maria Valentina Dinu*

Department of Functional Polymers, "Petru Poni" Institute of Macromolecular Chemistry, Grigore Ghica Voda Alley 41A, 700487, Iasi, Romania.
* Correspondence: vdinu@icmpp.ro

Figure S1. FTIR spectra of CG0.5GA5, CG1GA5 and CG2GA5 cryogels.
Figure S2. FTIR spectra of CG0.5GA7.5, CG1GA7.5 and CG2GA7.5 cryogels.
Figure S3. FTIR spectra of HG2GA10 sponge, before and after loading of CCM.
Figure S4. FTIR spectra of CG2GA5 and CG2GA7.5 sponges after loading of CCM.
Figure S5. SEM micrograph of HG2GA10 sponge (Magnification: 150x).
Table S1. Mean pore size of CS cryogels before and after loading of CCM.
Table S2. EDX elemental analysis of CS sponges, before and after loading of CCM.
Table S3. Compressive strength, maximum sustained compression and elastic moduli of CS sponges.
Figure S6. Maximum sustained compression (red bars) and compressive strength (blue bars) of CG2GA10 cryogels under cyclic stress-strain measurements.
Figure S7. (A) Optical images and (B) SEM micrographs of CG1GA10, CG2GA5, and CS2GA7.5 films obtained by applying a compressive force of 450 N at a rate of 1 mm/min.
Figure S8. The chemical structure of CCM (A) and optical images of CCM-loaded CS sponges (B).
Table S4. CCM loading efficiency and CCM loading capacity into CS sponges.

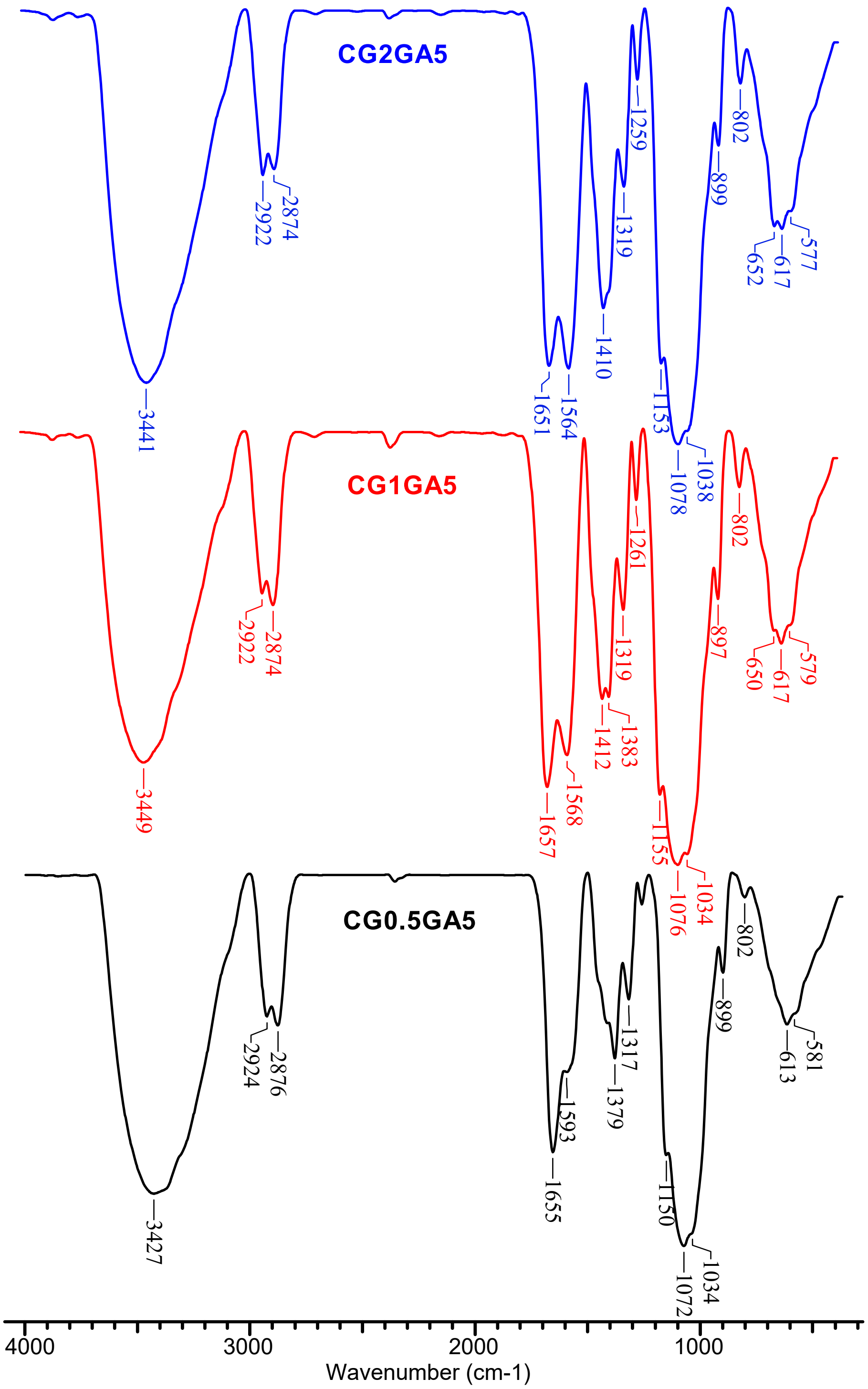

Figure S1. FTIR spectra of CG0.5GA5, CG1GA5 and CG2GA5 cryogels.

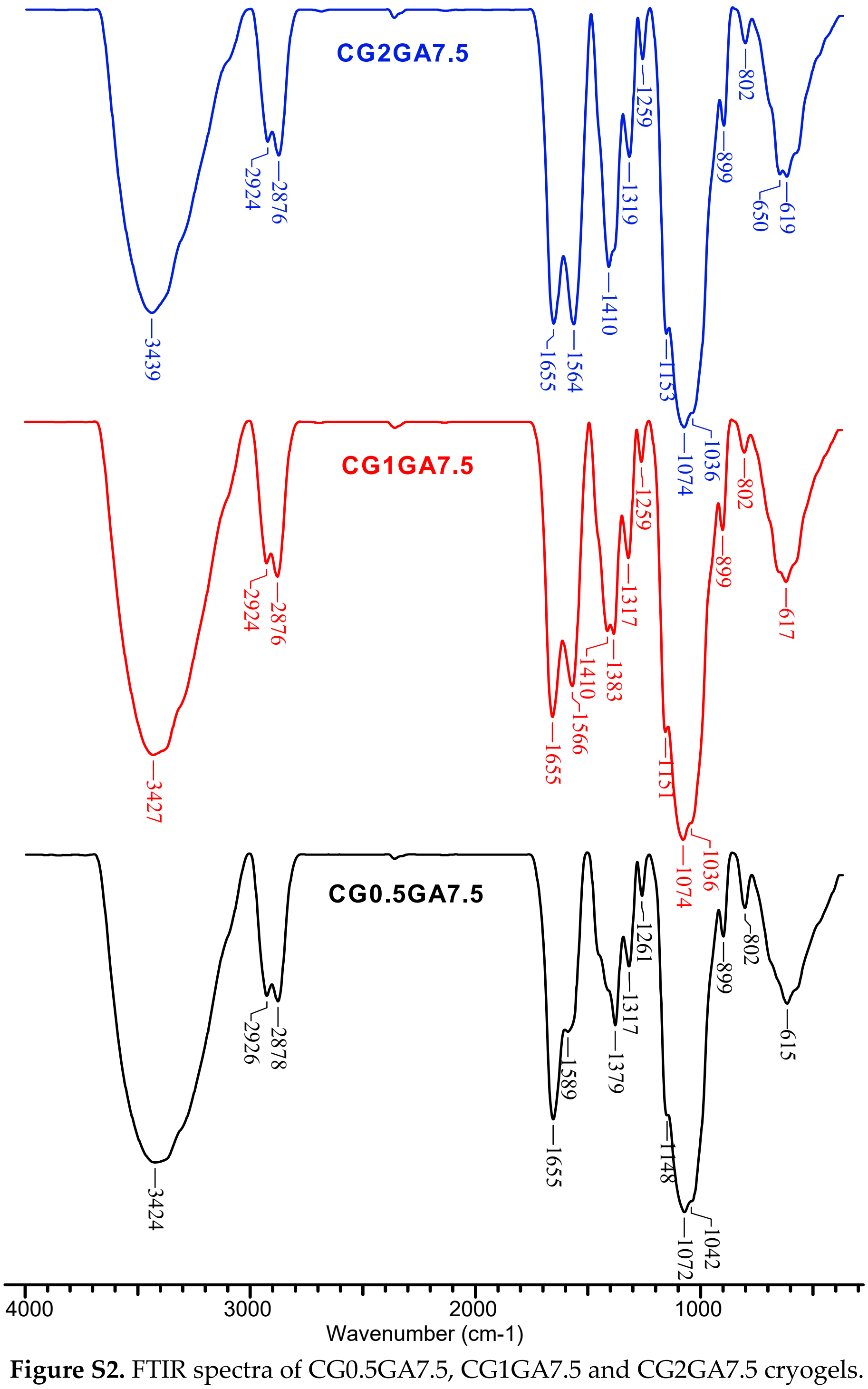

Figure S2. FTIR spectra of CG0.5GA7.5, CG1GA7.5 and CG2GA7.5 cryogels.

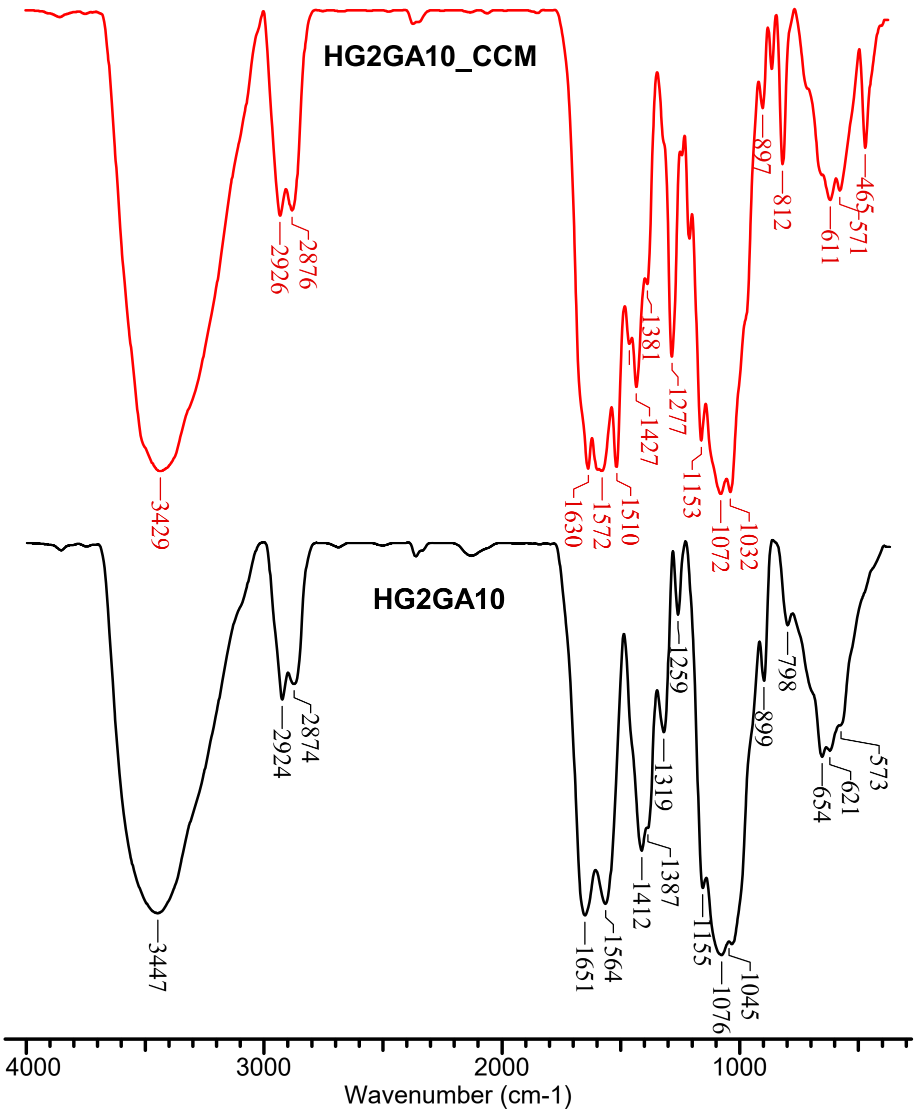

Figure S3. FTIR spectra of HG2GA10 sponge, before and after loading of CCM.

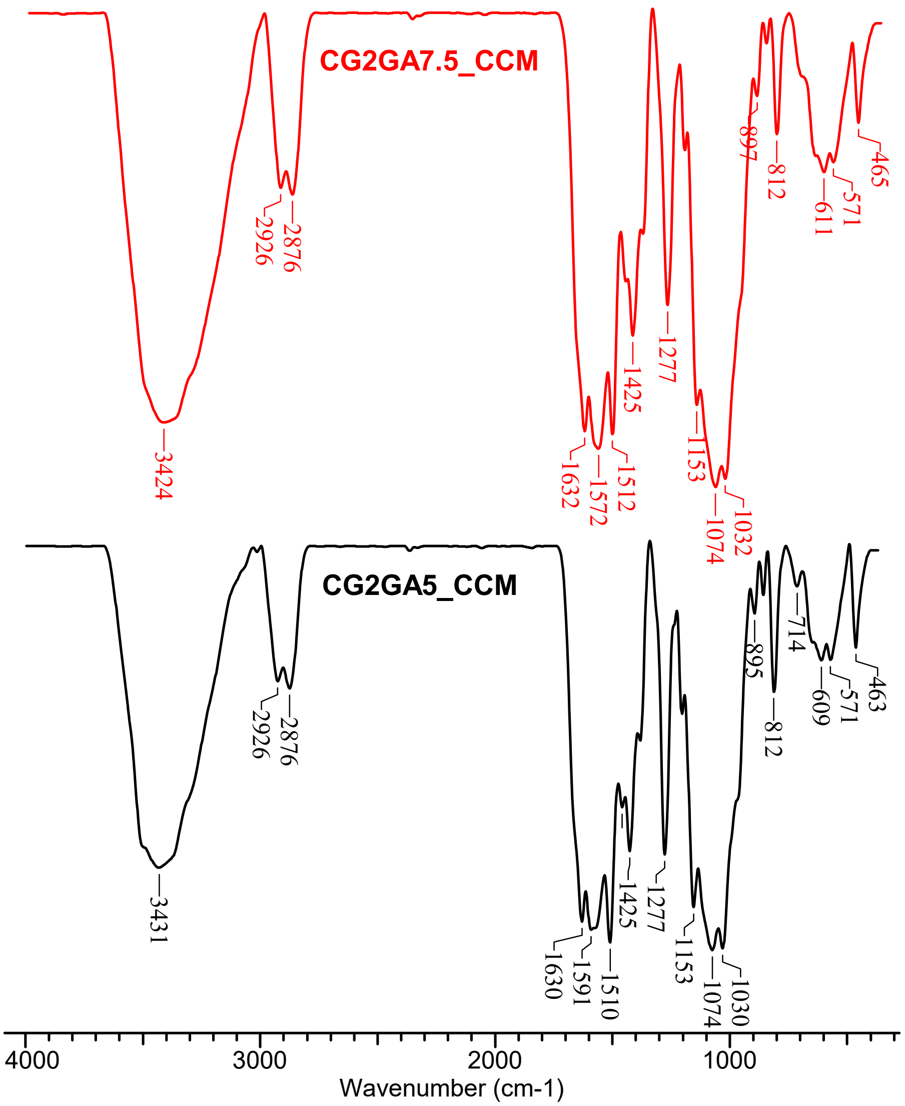

Figure S4. FTIR spectra of CG2GA5 and CG2GA7.5 sponges after loading of CCM.

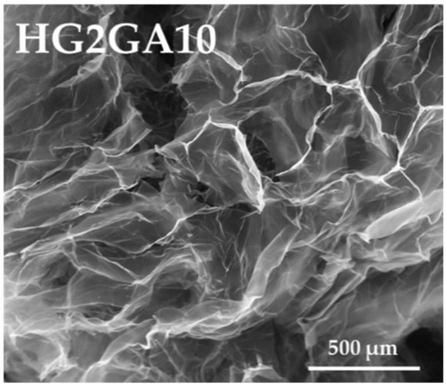

Figure S5. SEM micrograph of HG2GA10 sponge (Magnification: 150×).

Table S1. Mean pore size of CS cryogels before and after loading of CCM.

Sample	Mean pore size (μm)
CG0.5GA5	84.36
CG0.5GA7.5	74.12
CG0.5GA10	82.82
CG1GA5	75.85
CG1GA7.5	78.55
CG1GA10	76.54
CG2GA5	83.96
CG2GA7.5	58.33
CS2GA10	52.69
CG0.5GA10 + CCM	43.91
CG1GA10 + CCM	60.52
CG2GA5 + CCM	51.24
CG2GA7.5 + CCM	55.32
CG2GA10 + CCM	55.14

Table S2. EDX elemental analysis of CS sponges, before and after loading of CCM.

Sample	C, %	N, %	O, %
**CG0.5GA5**	67.90 ± 1.14	6.95 ± 1.40	25.13 ± 1.02
**CG0.5GA7.5**	67.04 ± 1.40	5.92 ± 0.72	27.02 ± 0.77
**CG0.5GA10**	68.62 ± 2.44	5.78 ± 0.59	25.62 ± 1.87
**CG1GA5**	62.62 ± 1.72	7.90 ± 0.57	29.48 ± 1.29
**CG1GA7.5**	63.70 ± 2.02	7.46 ± 0.85	28.84 ± 1.36
**CG1GA10**	63.40 ± 2.50	8.52 ± 1.51	28.06 ± 1.49
**CG2GA5**	63.42 ± 2.28	7.80 ± 0.76	28.80 ± 1.76
**CG2GA7.5**	63.38 ± 1.34	7.82 ± 0.68	28.80 ± 0.87
**CG2GA10**	63.78 ± 2.09	7.72 ± 1.11	28.48 ± 1.11
**HG2GA10**	60.12 ± 0.95	7.20 ± 0.45	32.70 ± 1.11
**CG0.5GA10 + CCM**	60.77 ± 1.14	7.23 ± 0.67	31.73 ± 0.59
**CG1GA10 + CCM**	57.5 ± 0.93	8.83 ± 0.09	33.47 ± 0.83
**CG2GA5 + CCM**	59.2 ± 1.90	7.80 ± 0.37	32.67 ± 1.52
**CG2GA7.5 + CCM**	56.60 ± 1.12	8.60 ± 0.42	34.57 ± 0.78
**CG2GA10 + CCM**	60.67 ± 1.61	7.37 ± 0.65	31.70 ± 0.99
**HG2GA10 + CCM**	56.33 ± 0.98	7.83 ± 0.37	35.70 ± 0.86

Table S3. Compressive strength, maximum sustained compression and elastic moduli of CS sponges.

Sample	Compressive strength, kPa	Maximum sustained compression, %	Elastic modulus, kPa
CG0.5GA5	-	-	-
CG0.5GA7.5	-	-	-
CG0.5GA10	-	-	-
CG1GA5	913.89 ± 43.01	83.08 ± 1.13	1.48 ± 1.01
CG1GA7.5	1007.32 ± 18.51	86.39 ± 1.26	3.96 ± 0.27
CG1GA10	1060.28 ± 22.97	87.74 ± 0.05	4.57 ± 0.63
CG2GA5	820.35 ± 63.61	87.86 ± 0.77	6.03 ± 1.73
CG2GA7.5	860.35 ±20.31	89.23 ± 0.82	11.21 ± 3.49
CG2GA10	865.96 ± 14.87	86.87 ± 1.10	12.53 ± 3.37
HG2CS10	1.47	32.00	-

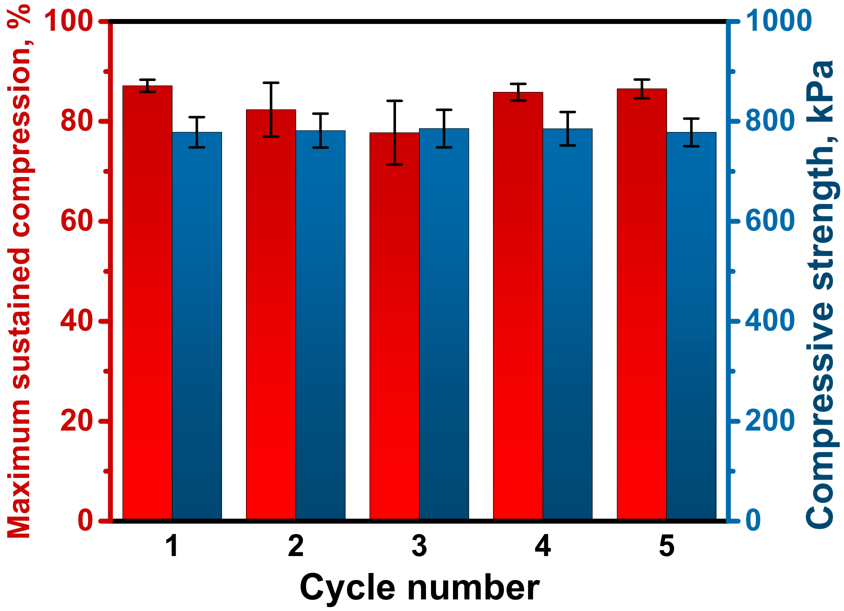

Figure S6. Maximum sustained compression (red bars) and compressive strength (blue bars) of CG2GA10 cryogels under cyclic stress-strain measurements.

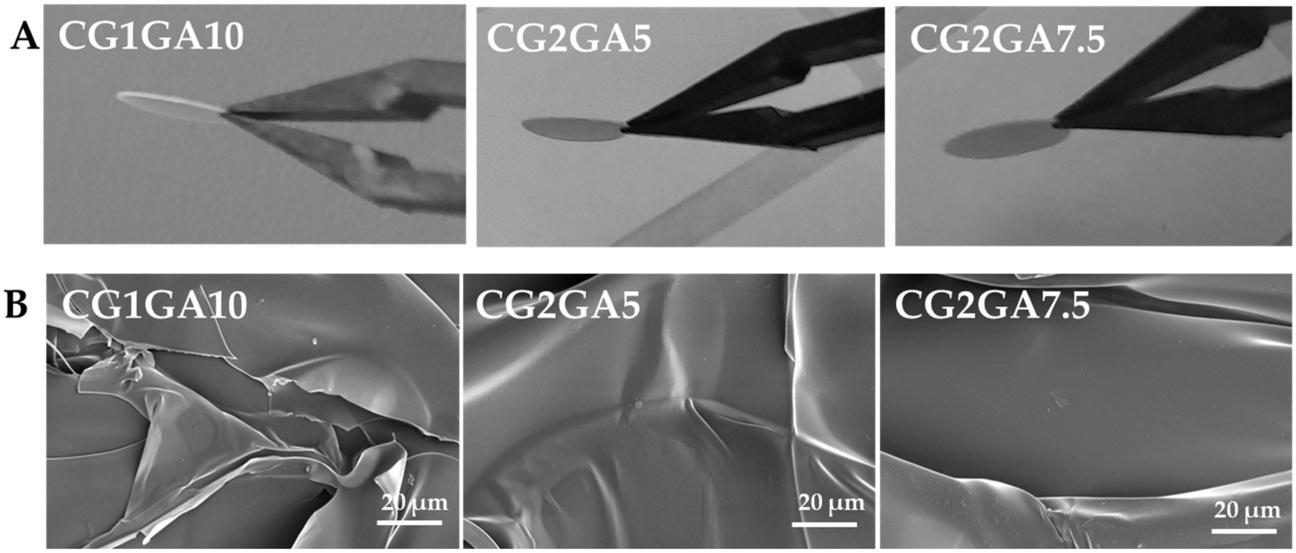

Figure S7. (A) Optical images and (B) SEM micrographs of CG1GA10, CG2GA5, and CS2GA7.5 films obtained by applying a compressive force of 450 N at a rate of 1 mm/min.

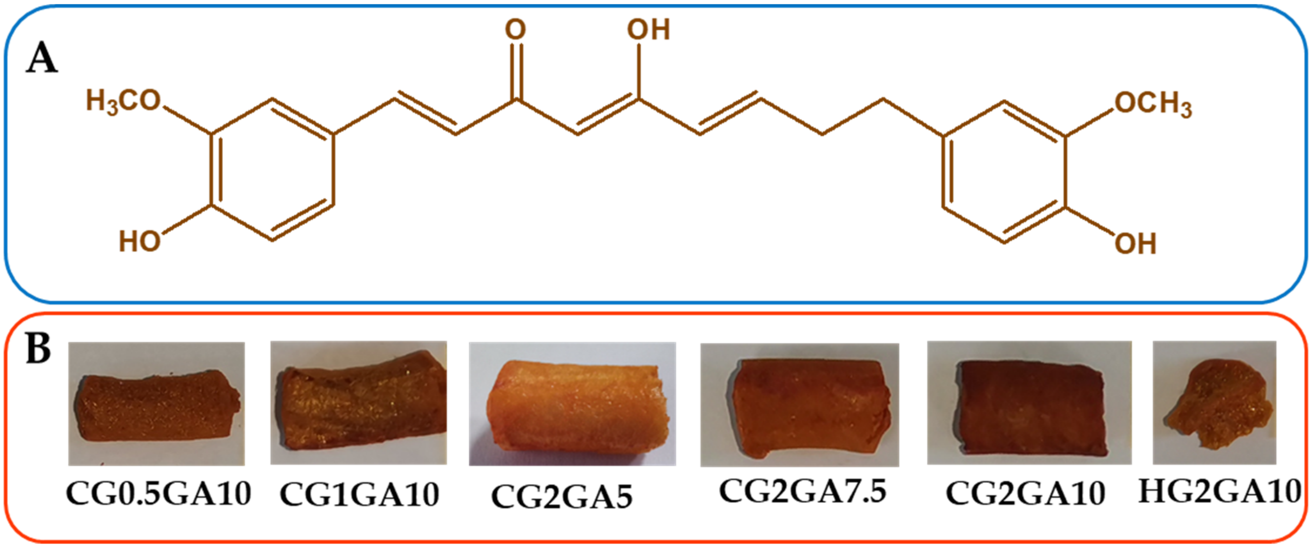

Figure S8. The chemical structure of CCM (A) and optical images of CCM-loaded CS sponges (B).

Table S4. CCM loading efficiency and CCM loading capacity into CS sponges.

Sample	LE, %	DL, %
CG0.5GA10	83.00	21.43
CG1GA10	83.28	17.65
CG2GA5	80.90	12.50
CG2GA7.5	92.33	19.48
CG2GA10	98.27	17.44
HG2CS10	87.37	18.99
